# Intra-cesarean insertion and fixation of frameless intrauterine devices

**DOI:** 10.4274/tjod.90532

**Published:** 2017-03-15

**Authors:** Ateş Karateke, Abdulkadir Turgut, Özkan Özdamar, Dirk Wildemeersch

**Affiliations:** 1 İstanbul Medeniyet University Faculty of Medicine, Department of Obstetrics and Gynecology, İstanbul, Turkey; 2 Gynecological Outpatient Clinic and IUD Training Center, Ghent, Belgium

**Keywords:** Intrauterine device, immediate contraception, frameless intrauterine devices

## Abstract

Various contraceptive methods are available to postpartum women including hormonal and nonhormonal barriers, as well as injectable forms. Of all the available birth control methods, intrauterine devices (IUD) are felt by many to be the near-ideal form of contraception, and are recommended by advocacy groups, physicians, and gynecological organizations worldwide. Immediate postpartum IUD insertion deserves greater attention because it can provide immediate contraception, prevents repeat unintended pregnancies, and may serve to reduce the incidence or need for secondary cesarean delivery; however, insertion of conventional T-shape IUDs immediately post placenta delivery is limited by their high expulsion and displacement rates. Anchoring of frameless-design IUDs that lack conventional cross-arms to the uterine fundal surfaces has been medically and commercially available throughout Europe for many years. The placement technique is simple, has minimal patient discomfort, and high long-term patient acceptance due to its high degree of uterine compatibility as a consequence of its small size and segmented design. Frameless-design IUD implantation appears to represent a major advance, suitable for general use, due to its lack of timing restraints and its simplicity of attachment, which only requires limited training.

## PRECIS:

Frameless design intrauterine device implantation appears to represent a major advance, suitable for general use due to its lack of timing restraints and its simplicity of attachment.

## INTRODUCTION

The ideal time for postpartum contraception either as a precautionary measure or as a family planning tool is immediately post-delivery. Immediate contraception is convenient and timely because a woman is actively evaluating her current and future family planning options. A woman’s return to fertility post-delivery is not always predictable because it can occur as soon as 3 weeks in non-lactating women and may not necessarily be accompanied by menses. The pregnancy environment represents the near-ideal timing for discussions with patients in need of contraception, the nature of the products available, and their individual benefit and risks. The patient’s receptiveness and willingness to select a given form of contraception is a critical component in allowing a woman to adequately manage her contraception needs.

Various contraceptive methods are available to postpartum women including hormonal and nonhormonal barriers, as well as injectable forms. Unfortunately, as reviewed by Trussell^(1)^, many of these methods although effective, have a high degree of failure when used imperfectly. For parous women, most physicians and the World Health Organization recommend an interdelivery interval of 18 to 24 months, because a second pregnancy too soon after the first could have detrimental effects on the woman herself, her ability to carry the baby to term, the viability of the infant, and its overall growth/development^(2)^. Women undergoing cesarean sections with an interdelivery interval shorter than 18 months have the added risk of possible uterine rupture^(3)^. Use of contraception as early as possible post-delivery would assure prevention of uterine rupture post-cesarean section, thus allowing for the wound to heal as well as for the woman to fully recover from her pregnancy. Effective contraception in these women will be valuable in reducing the risk of unintended pregnancies, and may allow for many women to have follow-on vaginal over cesarean deliveries.

Of all the available birth control methods, intrauterine devices (IUD) are felt by many to be the near-ideal form of contraception, and are recommended by advocacy groups, physicians, and gynecological organizations worldwide. IUDs have the advantage of high effectiveness as well as having an extremely low failure rate, in part because of the lack of involvement by the women. Immediate postpartum IUD insertion deserves greater attention because it can provide immediate contraception, prevents repeat unintended pregnancies, and may serve to reduce the incidence or need for secondary cesarean delivery^(4)^. Unfortunately, insertion of conventional T-shape IUDs immediately post placenta delivery is limited by their high expulsion and displacement rates^(5,6)^. A study conducted by Çelen et al.^(7)^ in Turkey in 2011 noted an expulsion rate of 17.6% at 12 months with the TCu380A IUD inserted immediately following cesarean section delivery. The inability of these devices to be retained clearly affects their effectiveness, but as importantly, overall patient acceptance. Women, especially those undergoing cesarean delivery, could benefit from immediate post-placenta IUD insertion because it would allow a sufficient period for the uterus, as well as the woman, to recover from surgery via a highly effective and long-lasting contraceptive. In these women, a low expulsion risk is therefore paramount with women having the added benefit of the IUD being easily reversible with a rapid return to fertility.

Over the past decades, attempts have been made to solve the expulsion problem encountered with conventional T-shape IUDs by modifying existing devices, such as adding absorbable sutures (delta-T) or additional appendages. These attempts were minimally successful. Expulsion rates varying from 5% at 12 months to up to 50%, and even higher if partial expulsions are included, have been reported^(8,9,10)^.

Timing of insertion post-placenta delivery is of critical importance with T-shape devices. Studies have shown that if inserted at times beyond 10 minutes post-delivery, expulsion rates were higher than those observed in normal women.

**Figure 1:**
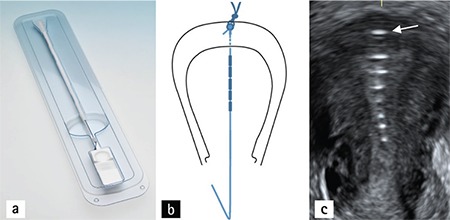
a) Insertion apparatus for the insertion of the frameless intrauterine devices following cesarean section delivery b) Illustration of the anchoring technique; a biodegradable suture holds the anchor in place in the uterine fundus until involution c) Coronal ultrasound of the position of the frameless intrauterine devices after involution of the uterus with an anchor marker in the fundus (arrow)

Anchoring of frameless design IUDs that lack conventional cross-arms to the uterine fundal surfaces has been medically and commercially available throughout Europe for many years in the form of GyneFix (Contrel Research, Belgium). The placement technique is simple, has minimal patient discomfort, and high long-term patient acceptance due to its high degree of uterine compatibility as a consequence of its small size and segmented design. Since its inception, the technology has passed through several phases of improvement, design modifications, and clinical testing intended to maximize patient comfort and tolerability producing 5-year continuation rates in excess of 90%. Clinical studies were also conducted to evaluate the impacts of immediate insertion of a frameless IUD during cesarean section, as well as after vaginal delivery, on the bleeding pattern, duration of lochia, and healing of uterus. Accordingly, no significant difference in postpartum hemorrhage, continuance of lochia, and healing of uterus, was observed^(11)^. A novel minimally invasive surgical approach was devised for suspending the frameless copper IUD for intra-cesarean insertion, which takes advantage of the full visualization and access to the uterus that is achieved during cesarean delivery. The technique consists of the precise placement of the anchoring knot immediately below the serosa of the uterine fundus, followed by fixing the knot in place with a conventional absorbable suture (Figure 1a, 1b). In several weeks, the uterus regains its normal tonicity, the suture is absorbed, and the anchor retained as seen in women undergoing conventional interval insertion. The procedure is simple and can be performed at any convenient post-delivery period, and takes less than 4 minutes with no discomfort to the patient and minimal surgical risk. The IUD tail is looped in the cervical canal or is cut at the level of the cervix. The anchoring technique has been shown to be easy, quick and safe in a pilot trial with no expulsions at 12 months. Ongoing studies conducted in Turkey confirm the efficacy of the technique and high acceptability of the frameless IUD by women. To check IUD placement, a follow-up sonography can be performed to localize the stainless steel marker attached to the anchoring knot (Figure 1c). Removal of the IUD is similar to the removal after interval insertion of the device accomplished by simply pulling on the IUD string. In the event that the tail is in the cavity, it is accessible by using thin alligator forceps (3 mm) if/when removal is requested. The copper releasing frameless IUD and inserter, which were specifically designed to facilitate use immediately post-placental delivery after cesarean deliveries, is now available in Turkey. Conventional insertion of frameless IUDs in normal women is already available in Turkey. The developers are also finalizing the development of a levonorgestrel-releasing frameless system, which may have additional advantages in some women.

Frameless-design IUD implantation appears to represent a major advance, suitable for general use due to its lack of timing restraints and its simplicity of attachment, which only requires limited training. It affords the patient and her physician additional options for contraceptive control that may likely serve to reduce the number and frequency of unintended pregnancies. Frameless IUDs appear to have advantages over framed T-shaped IUDs because the latter may cause discrepancy with the uterine cavity and embedment during involution of the uterus, particularly during prolonged lactation as hyperinvolution in these women is not uncommon^(12)^.

### Disclosure

Dr. Dirk Wildemeersch has been involved in the development of novel contraceptive systems for use in the uterus. He is currently an advisor in devising new concepts in controlled release for contraception and gynecological treatment.
